# A User’s Guide to the *ALiEM Emergency Medicine Match Advice* Web Series

**DOI:** 10.5811/westjem.2017.3.33841

**Published:** 2017-05-01

**Authors:** Michael A. Gisondi, Abra Fant, Nahzinine Shakeri, Benjamin H. Schnapp, Michelle Lin

**Affiliations:** * Northwestern University Feinberg School of Medicine, Department of Emergency Medicine, Chicago, Illinois; † University of Wisconsin School of Medicine and Public Health, Department of Emergency Medicine, Madison, Wisconsin; ‡ University of California San Francisco, Department of Emergency Medicine, San Francisco, California

## Abstract

*ALiEM EM Match Advice* is a web series hosted on the Academic Life in Emergency Medicine website. The intended audience includes senior medical students seeking a residency in emergency medicine (EM) and the faculty members who advise them. Each episode features a panel of three EM program directors who discuss a critical step in the residency application process. This article serves as a user’s guide to the series, including a timeline for viewing each episode, brief summaries of the panel discussions, and reflection questions for discussion between students and their faculty advisors.

## INTRODUCTION

Emergency medicine (EM) is a competitive specialty. The National Residency Matching Program^®^ (NRMP) offered 1,895 positions in EM in the 2016 Main Residency Match^®^ (the Match), with a fill rate of 99.9% overall and 78.4% by allopathic U.S. senior medical students.^1^ The residency application process is a stressful time for medical students who must make difficult decisions about selecting away rotations, submitting applications, scheduling interviews, and creating a rank order list. Medical students access a variety of sources of information for advice about the Match process. In addition to their faculty advisors, students consult anonymous online blog sites, near-peers, and each other – all sources with variable expertise and information quality.

In 2014 Academic Life in Emergency Medicine (ALiEM) launched *EM Match Advice*, a video-based web series designed to assist senior medical students in their preparation for the Match process in EM. Each episode features a discussion of an important aspect of the Match by a panel of three EM program directors (PD). Episodes are recorded using Google Hangout on Air^®^ and archived on YouTube™. These have since been converted to podcasts offered on SoundCloud™. A different panel of PDs is invited for each recording to present diverse opinions that reflect the variety of training opportunities available to EM-bound students across the country. To date, there are 12 episodes in the series with contributions by 36 current or recent EM PDs.

This article will briefly review the content of each episode of *ALiEM EM Match Advice* in the form of a user’s guide for students and EM faculty advisors who may freely access this web series online.

## EPISODE SUMMARIES

### 1. Is Emergency Medicine Right for You?

URL: https://www.aliem.com/2016/em-match-advice-emergency-medicine-right/

Publication Date: September 7, 2016

Panelists: Larissa Velez (University of Texas Southwestern), Brian Levine (Christiana Care), Michele Dorfsman (University of Pittsburgh)

#### Summary

The panelists reflect on their experiences practicing EM, highlighting the key characteristics of emergency physicians (EP), the best parts of the profession, and the challenges to be expected of a career in EM.

EPs tend to have broad clinical interests and often enjoy most core clinical rotations as medical students. They are team-oriented, like variety in their practice, prefer being busy while at work, are comfortable making quick decisions with limited information, and gain satisfaction from brief, intense relationships with patients. Highlights of the profession include caring for patients of all ages, socioeconomic groups and cultural backgrounds and acquiring expertise in resuscitation, acute care, and life-sustaining procedures.

Students should be cautioned that a career in EM is not simply an easy lifestyle choice. High-intensity shifts can be exhausting, and burnout is real. Switching between day and night shifts is physically challenging. EM is not “all action” and students should anticipate that resuscitations comprise only a small percentage of the cases on an average shift.

#### Reflection Questions

- Which tasks do I look forward to performing while at work?
- Am I uncomfortable with uncertainty?
- How do I deal with interruptions and task-switching?

### 2. VSAS 101: Securing an Away EM Rotation

URL: https://www.aliem.com/2015/em-match-advice-vsas-101/

Publication Date: May 22, 2015

Panelists: Susan Stroud (University of Utah), Cullen Hegarty (Regions Medical Center/HealthPartners), Scott Sherman (Stroger/Cook County Hospital)

#### Summary

The panelists discuss the process of securing EM away rotations using the AAMC Visiting Student Application Service^®^ (VSAS). Rotating at a program away from one’s home institution is a valuable experience that can provide exposure to a different style of EM and may even strengthen a student’s residency application. In addition, many residency programs expect to review grades and evaluations from an applicant’s home and away EM rotations prior to extending them an interview invitation.

In this episode, the panelists describe the key elements of a VSAS application, including additional or supplemental application materials that may be requested. Logistics such as application fees and timelines are reviewed. Students should work with their advisors and EM clerkship directors when preparing their VSAS application and should be as flexible as possible when selecting schools and rotation dates in VSAS.

Some schools do not participate in VSAS and must be contacted directly. It is very difficult for students at medical schools outside the U.S. to obtain away rotations in EM; international students should focus their applications on schools that partner with their own medical school, if such partnerships exist.

#### Reflection Questions

- Will an away rotation in EM strengthen my application? How many away rotations should I do?
- How can I use my EM away rotation(s) to help me decide which type of residency program is right for me?
- How should I schedule the clerkships in my fourth year of medical school?

### 3. The EM Rotation

URL: https://www.aliem.com/2014/em-match-advice-em-rotation-eras-competitive/

Publication Date: August 20, 2014

Panelists: Lainie Yarris (Oregon Health and Sciences University), Maria Moreira (Denver Health), Jan Shoenberger (LAC-USC)

#### Summary

In this episode, panelists describe the honors-level performance on the EM rotation. Students should strive to function like an intern by being self-directed, taking ownership of their patients, and anticipating the needs of their team. It is important to maintain a friendly, humble, upbeat, and professional demeanor. Students should understand the criteria on which they are being graded, and should keep in mind that they are being assessed at all times during the rotation.

#### Reflection Questions

- What does it mean to “function like an intern?”
- What does it mean to “pass the 3AM test?”
- How can I balance being professional and likeable?

### 4. ERAS: Electronic Residency Application Service

URL: https://www.aliem.com/2014/em-match-advice-em-rotation-eras-competitive/

Publication Date: August 20, 2014

Panelists: Gene Hern (Alameda County - Highland), Laura Hopson (University of Michigan), Joshua Broder (Duke University)

#### Summary

The panelists discuss key considerations for completing the residency application.

Standardized letters of evaluation (SLOEs) for EM, EM rotation grades, and the Medical Student Performance Evaluation (MSPE) tend to be of highest importance to programs when selecting which students to interview, while the personal statement is generally less important.^2^–^4^

The application should craft a narrative about the student and demonstrate a high attention to detail. Address any file irregularities or potential red flags carefully and in consultation with a trusted and experienced advisor.^5^ During interviews, applicants should be prepared to discuss any item that was included on their application.

#### Reflection Questions

- What narrative does my application tell?
- What are the weaknesses of my file, and how can I address these in my application?
- What details of my application are interviewers most likely to ask about?

### 5. Mirror, Mirror on the Wall: Am I Competitive?

URL: https://www.aliem.com/2014/em-match-advice-em-rotation-eras-competitive/

Publication Date: August 20, 2014

Panelists: Andrew Perron (Maine Medical Center), Madonna Fernandez-Frackelton (Harbor-UCLA), Kevin Biese (University of North Carolina, Chapel Hill)

#### Summary

In this episode, panelists discuss the importance of accurately assessing a student’s application in order to help them apply to an appropriate number and mix of residency programs. Students should seek feedback on their competitiveness from a reliable source such as the residency PD, clerkship director, or a trusted advisor in EM who has knowledge about the Match process. Peers and near-peers do not have the expertise to accurately assess an applicant’s competitiveness.

The number of residency program applications recommended for a given student depends on the overall competitiveness of their file, any geographic preferences for training, and participation in the Match paired with another applicant (i.e., “Couples Match”). Most applicants should consider applying to approximately 30 programs; highly competitive applicants should apply to fewer programs, and less competitive applicants or those in the Couples Match may need to apply to more programs.^1^,^6^–^8^

#### Reflection Questions

- What are the strengths of my application? Which programs are likely to value those strengths most?
- Based on my overall competitiveness, to how many programs should I apply?

### 6. The Non-LCME Applicant

URL: https://www.aliem.com/2015/em-match-advice-series-the-non-lcme-applicant/

Publication Date: September 16, 2015

Panelists: Merle Carter (Einstein Healthcare), Doug Finefrock (Hackensack University Medical Center), Damon Kuehl (Virginia Tech Carilion)

#### Summary

This episode highlights the special considerations of “non-LCME applicants” entering the allopathic EM Match. The non-LCME applicant is defined as any candidate not in their final year of training at an LCME-accredited medical school in the U.S., Puerto Rico, or Canada.^9^ The episode is divided into segments that address the specific considerations of osteopathic applicants, international applicants, and military applicants, and offers pearls and pitfalls for each.

Approximately 20% of EM positions available in the Match are filled by non-LCME applicants. One key recommendation for the non-LCME applicant is to “make your application look like that of an LCME applicant”: take the USMLE examinations, seek away EM rotations at hospitals with allopathic EM residency programs, and attempt to obtain letters of recommendation written in the SLOE format.

#### Reflection Questions

- For osteopathic students: Will I apply to the allopathic match, osteopathic match, or both?
- As a non-traditional applicant, how can I highlight my unique characteristics and life experiences?
- How do I identify programs that regularly match non-LCME applicants?

### 7. Program Directors Reflect on the 2015 Match

URL: https://www.aliem.com/2015/em-match-advice-reflections-from-the-2015-em-residency-match/

Publication Date: June 27, 2015

Panelists: Francis DeRoos (University of Pennsylvania), Megan Boysen Osborn (University of California, Irvine), Jason Wagner (Washington University in St. Louis)

#### Summary

In the 2015 Match, EM had a 79% fill rate by LCME seniors and very few positions available through the NRMP SOAP^®^ (Supplemental Offer and Acceptance Program), making EM a moderately competitive specialty.^10^ Panelists in this episode discuss their impressions of the 2015 EM Match.

One emerging trend has been an increase in the number of applications per applicant.^10^ Applicants should consult with their EM advisor for a recommendation on the appropriate number of applications to submit. Avoid over-applying and over-interviewing; these approaches are costly and do both applicants and programs a disservice. Students should cancel scheduled interviews early if they are no longer interested in interviewing at a program in order to release these spots for other applicants.

#### Reflection Questions

- How competitive is my file?
- How many applications should I submit?

### 8. Program Directors Reflect on the 2016 Match

URL: https://www.aliem.com/2016/em-match-advice-reflect-2016/

Publication Date: July 10, 2016

Panelists: Michael Bond (University of Maryland), Christopher Doty (University of Kentucky), Diane Rimple (University of New Mexico)

#### Summary

Panelists discuss the 2016 EM Match and offer advice to future applicants. One continuing trend in 2016 is an increase in the number of applications per applicant; as in the previous episode, “Program Directors Reflect on the 2015 EM Match,” the panel emphasizes that applicants should refer to an EM advisor for a recommendation on the correct number of applications to submit based on their competitiveness.^1^ Panelists emphasize that there is no objective, meaningful ranking of EM residency programs endorsed in our specialty; applicants must create their own customized list of highly desirable programs based on what attributes they personally value.

#### Reflection Questions

- What do I value most in a potential residency program?
- From which faculty members should I request letters of recommendation?

### 9. Interviewing Strategies

URL: https://www.aliem.com/2014/em-match-advice-interviewing-strategies/

Publication Date: August 29, 2014

Panelists: Christine Babcock (University of Chicago), Linda Regan (Johns Hopkins University), Philip Shayne (Emory University)

#### Summary

The panelists discuss interview scheduling, preparation for the interview day, and specific dos and don’ts of the interview trail.^11^–^13^ Interviews should be scheduled as soon as possible after receiving an invitation. Based on NRMP data, applicants should aim for a goal of 10–14 interviews to maximize the probability of matching. More interviews may be necessary for a less competitive applicant or for the Couples Match.^14^

On interview day, applicants should be prepared to discuss their strengths and weaknesses, their reasons for pursuing EM, why they have chosen to interview at a particular residency program, and how they have dealt with situations that presented challenges or conflict. Applicants should come prepared with questions tailored to each type of interviewer; for example, ask resident interviewers questions about faculty teaching and ask the program director “big picture” questions about the program’s mission or future.

Do: act professionally in all interactions and situations, be engaged, have thoughtful questions for interviewers, and take notes after your interview day. Do not: cancel an interview with less than two weeks notice, speak negatively about other programs, or feel obligated to answer a Match-illegal or inappropriate question such as marital status or family planning.^15^

#### Reflection Questions

- How will I prepare for each interview day?
- How should I respond to an illegal or inappropriate interview question?
- What questions will I ask a junior resident? A senior resident? A faculty member? The program director? The department chair?

### 10. Post Interview Communications

URL: https://www.aliem.com/2014/em-match-advice-post-interview-communications/

Publication Date: November 15, 2014

Panelists: James Colletti (Mayo Clinic), Jessica Smith (Brown University), Jeff Schneider (Boston Medical Center)

#### Summary

The panelists discuss the etiquette of post-interview communication between applicants and programs. Programs must adhere to the NRMP Code of Conduct, which stipulates that post-interview communication to applicants must not be disingenuous or coercive.^16^–^18^ Likewise, it is essential for the applicant to protect and nurture their professional identity by ensuring that all communications with programs are courteous, polite and honest.

The panelists agreed that, although not all programs will be expecting it, it is generally a good idea to write genuine, content-specific thank you notes or e-mails to the PD and coordinator(s) from each program. It is appropriate for an applicant to reach out after their interview to obtain more information from the PD, faculty or residents; this type of post-interview communication is encouraged and likely has no effect on rank list position.^19^, ^20^ Similarly, returning to a program for a “second look” visit may be arranged through the program coordinator but is unlikely to have any effect on the candidate’s position on the rank list. Avoid inundating the PD or other program representatives with excessive e-mail correspondence.

Panelists agree that it is generally unnecessary to inform programs of how highly they will be ranked on an applicant’s rank order list, though it may be advantageous to share such information with a student’s number one choice. Such statements should only be made honestly; EM is a small community and integrity is vital to an applicant’s professional identity and future success.

#### Reflection Questions

- How will I communicate with programs after interview day?
- How can I develop and protect my professional identity?
- Should I return to a program for a second look?

### 11. Making the Perfect Rank Order List

URL: https://www.aliem.com/2014/em-match-advice-making-perfect-rank-order-list/

Publication Date: October 7, 2014

Panelists: Colleen Roche (George Washington University), Jonathan Davis (Georgetown University), Brian Stettler (University of Cincinnati)

#### Summary

The panelists discuss important considerations for creating a rank order list, including how the Match algorithm works and an approach to synthesize and prioritize the information obtained on interview day.

The Match algorithm favors the applicant. There is no way to “game” the system, so the best way for an applicant to structure the rank order list is to place programs in exact order of preference.^21^

It is easy to be overwhelmed by specific program details on interview day.^22^ Focus instead on the program’s “3 Ps:” its overarching philosophy, passion, and people. Applicants should ask themselves: Will this program inspire me to come to work? Will my career goals be supported? Which interview day excited or inspired me the most?

Although geography is an important consideration, avoid compromising the right fit and best educational experience for geography alone.^23^–^25^ Imagine opening the envelope on Match day: which program name is most exciting to see on the paper inside?

#### Reflection Questions

- What are my career goals? Which program or programs will support these goals?
- What are the program attributes that are most important to me?

### 12. What if I Don’t Match? What Is the SOAP?

URL: https://www.aliem.com/2016/em-match-advice-what-if-i-dont-match-what-is-the-soap/

Publication Date: January 17, 2016

Panelists: Daniel Egan (Mt. Sinai St. Luke’s-Roosevelt), Tiffany Murano (Rutgers University New Jersey Medical School), Mary Westergaard (University of Wisconsin)

#### Summary

In this episode, panelists discuss the logistics of the NRMP Supplemental Offer and Acceptance Program^®^ (SOAP) and the options available to an applicant who does not match into an EM residency program. The SOAP is a service of the NRMP that helps place unmatched applicants into unfilled residency slots.^26^ Applicants will learn if they matched on the Monday of Match Week, and they should work with their medical school to navigate the SOAP process if unmatched. Unfortunately, very few, if any, EM positions are available through the SOAP.

Students who do not match should reflect on the following questions in consultation with an EM advisor: Was there a deficiency in my ERAS file (poor grades on clinical rotations, negative comments on letters of recommendation, low USMLE scores)? Was my list of programs too competitive? Did I apply broadly enough? Did I interview poorly?

Unmatched applicants who want to apply to EM the following year have three immediate options to consider: (1) extend medical school by one year and reapply during the next Match cycle, (2) spend a year pursuing a graduate degree or a research project rather than entering internship, or (3) enter the SOAP for a preliminary, categorical or transitional year in another specialty. Be sure to address any application deficiencies during this time.

#### Reflection Questions

- Am I at risk of not matching into EM and why?
- What is my backup plan in the event that I do not match?

## Figures and Tables

**Figure:**
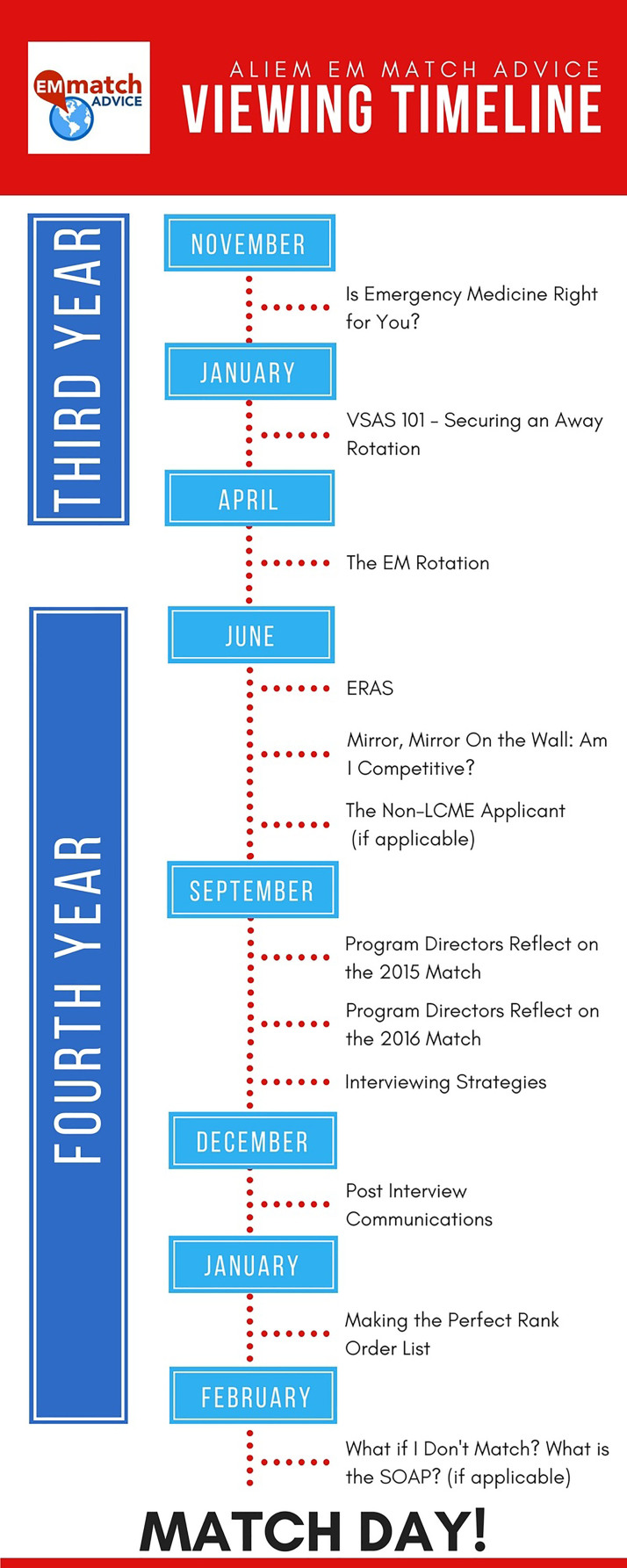
*ALiEM EM Match Advice* viewing timeline.
